# Effect of layer thickness and annealing temperature on the electrocatalytic activity of CNT/Pt counter electrode for triiodide reduction

**DOI:** 10.1016/j.dib.2018.08.127

**Published:** 2018-08-31

**Authors:** Van-Duong Dao, Ho-Suk Choi

**Affiliations:** a Theoretical Physics Research Group, Advanced Institute of Materials Science, Ton Duc Thang University, Ho Chi Minh City, Vietnam; b Faculty of Applied Sciences, Ton Duc Thang University, Ho Chi Minh City, Vietnam; c Department of Chemical Engineering and Applied Chemistry, Chungnam National University, Daejeon 305-764, South Korea

**Keywords:** CNT/Pt, Counter electrode, Dye-sensitized solar cell, Thickness, Temperature

## Abstract

The data presented in this article are related to the research article entitled “Balance between the charge transfer resistance and diffusion impedance in a CNT/Pt counter electrode for highly efficient liquid-junction photovoltaic devices” (Dao and Choi, 2018) [1]. This article presents the effect of annealing temperature and thickness of CNT/Pt film on the electrocatalytic activity of CNT/Pt counter electrode for triiodide reduction. For this purpose, we firstly fabricated CNT/Pt paste with different amount of CNT/Pt. The CNT/Pt film is then fabricated by doctor blade method.

**Specifications table**

**Table:** 

Subject area	*Physics, Chemistry*
More specific subject area	*Counter electrode of dye-sensitized solar cells*
Type of data	*Tables, Figures, Image, Text file*
How data was acquired	*Evaluating the electrochemical catalytic activity of electrodes:*
	*The redox behaviors of the electrodes under study were evaluated through a comparative analysis of their cyclic voltammograms (CVs) using three-electrode electrochemical cells. A Pt mesh and AgCl/Ag electrodes served as the CE and the reference electrode, respectively. A solution of 10 mmol LiI, 1 mmol I*_*2*_*and 100 mmol LiClO*_*4*_*was used as the electrolyte. The CVs were recorded in a range of 200 to − 500 mV at a scan rate of 100 mV s*^−^^*1*^.
	*Electrochemical impedance spectroscopy (EIS) was carried out with symmetrical dummy cells fabricated from two identical electrodes with a frequency range of 100 kHz to 100 mHz and a perturbation amplitude of 10 mV. The obtained spectra were fitted using the Z-view software (2.8d, Scribner Associates, Inc.) with reference to the proposed equivalent circuit.*
Data format	*Raw, filtered, analyzed*
Experimental factors	*CNT/Pt content, thickness, temperature*
Experimental features	*A solution of 40 mg ethyl cellulose in 900 mg alpha-terpineol was prepared, after which 10, 50, 100 and 200 mg of CNT/Pt powders were added to the prepared solution. In order to obtain the CNT/Pt paste, the mixture was ground through a three-roll mill. To realize the CNT/Pt film-coated FTO glass, the doctor-blade method was applied and the film was dried at 300 °C for 30 min.*
Data source location	*Chungnam National University, Daejeon, South Korea*
Data accessibility	*The data are available with this article.*

**Value of the data**

• CNT/Pt paste with different amount of CNT/Pt is carefully described.
• The reduction rate of triiodide ions increases with an increase in the amount of CNT/Pt in the paste.
• The electrocatalytic activity increases with an increase in the thickness of CNT/Pt film.
• The annealing temperature does not affect to electrocatalytic activity.
• Adhesion test indicates the stability of the counter electrodes.

## 1 Data

**Scheme 1:**
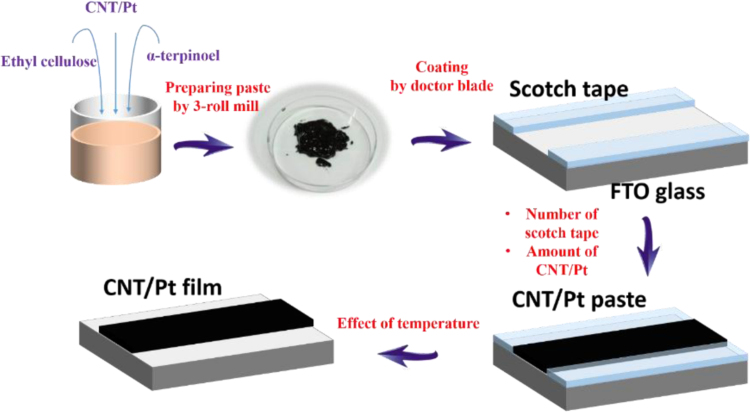
The fabrication process of making CNT/Pt paste and coating CNT/Pt on FTO glass substrate.

**Fig. 1:**
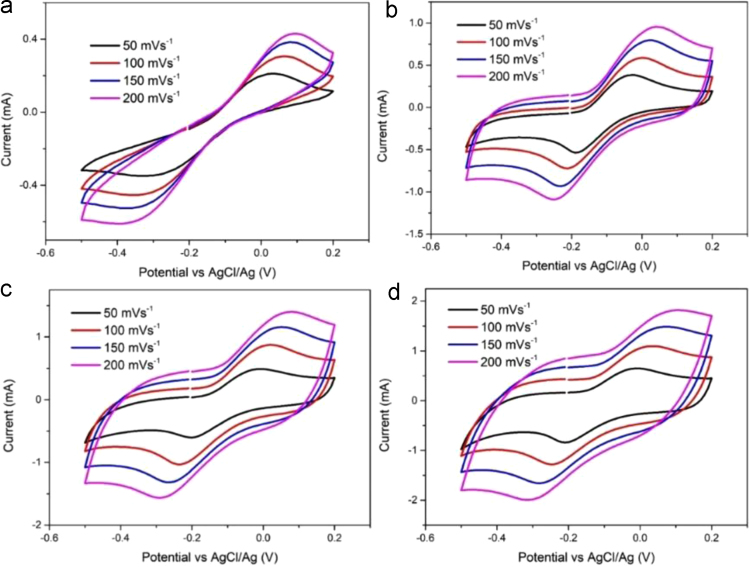
CV of a CNT/Pt CE prepared with 10 (a), 50 (b), 100 (c) and 200 (d) mg CNT/Pt with different scan rates.

**Fig. 2:**
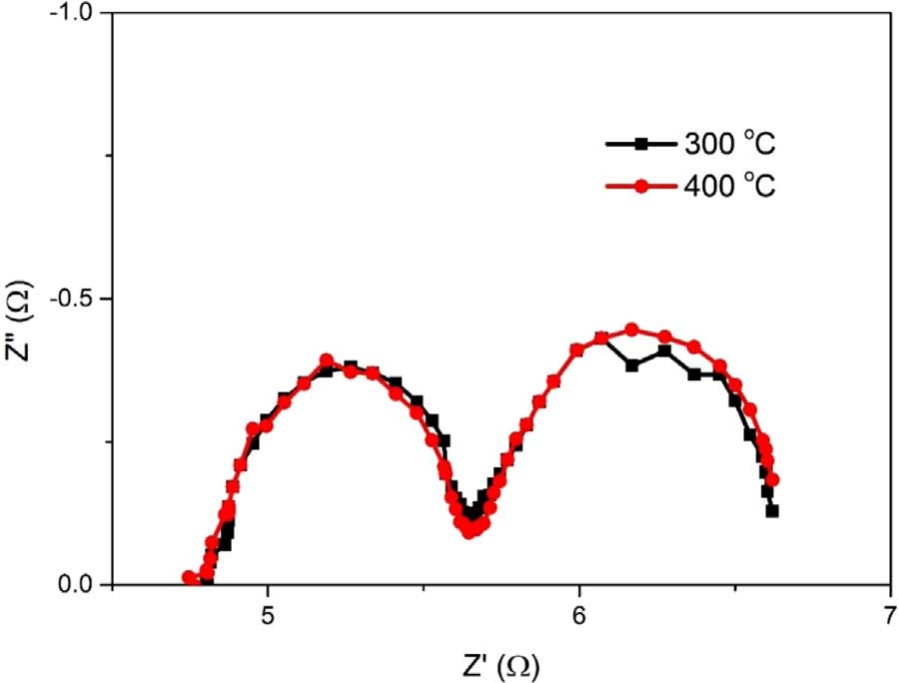
Nyquist plots of symmetrical dummy cells with two identical CEs prepared with different annealing temperatures.

**Fig. 3:**
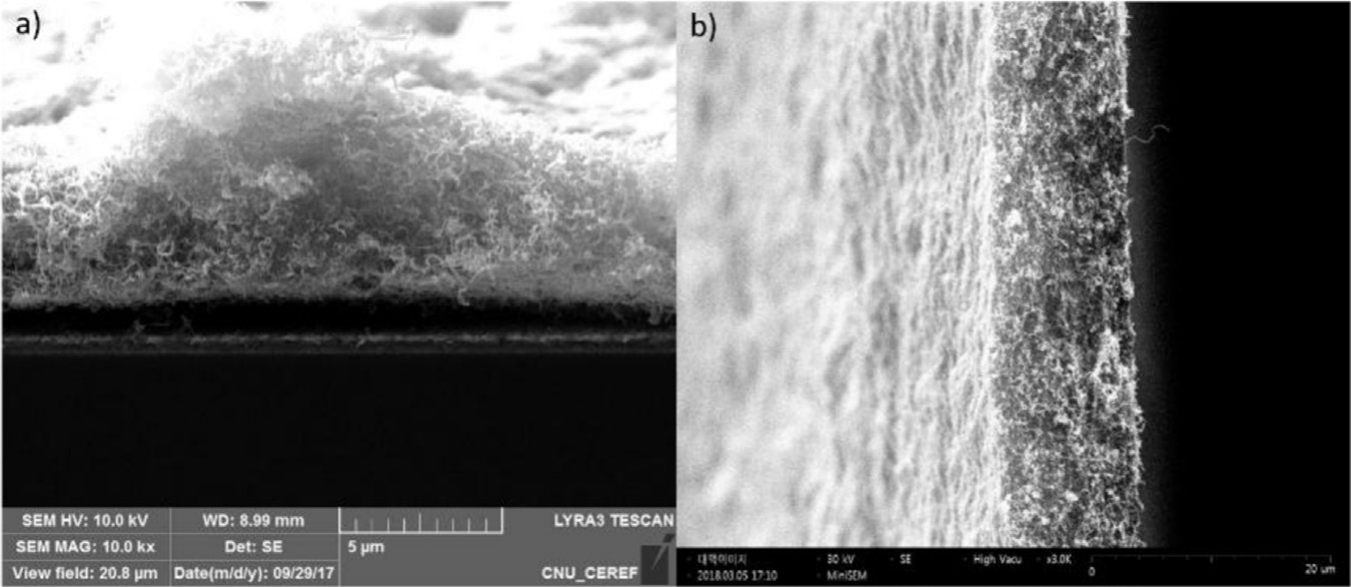
SEM images of 50 mg CNT/Pt paste coated on FTO glass with different thickness: a) 3.2 µm and b) 12 µm.

**Fig. 4:**
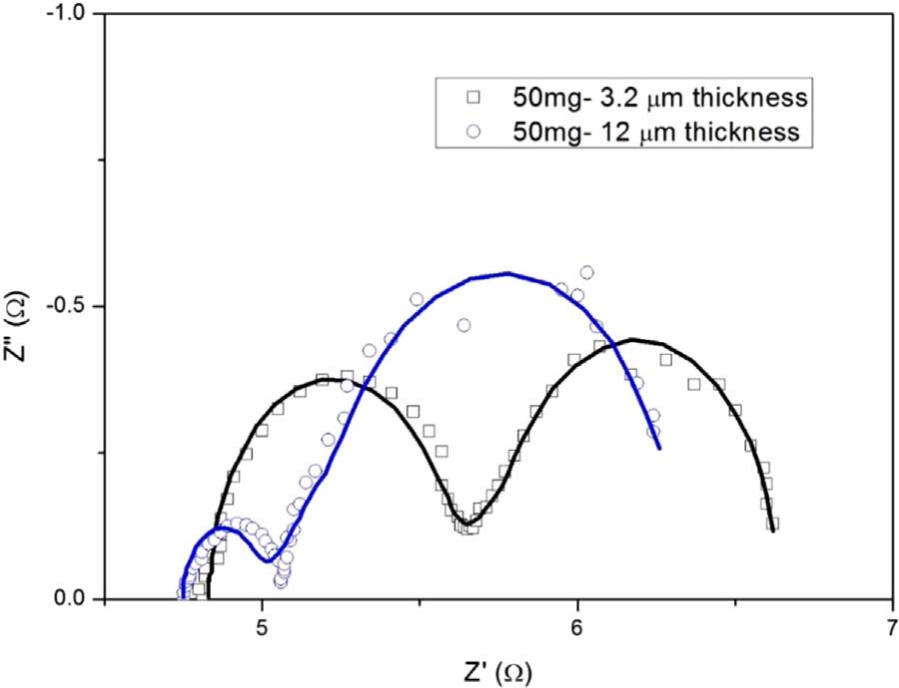
Nyquist plots of symmetrical dummy cells with two identical CEs prepared with different thickness.

The dataset of this article provides information on the effect of annealing temperature and thickness of CNT/Pt on the reduction of triiodide ions at counter electrode of dye-sensitize solar cells. Scheme 1 presents the fabrication process of preparing CNT/Pt paste and coating CNT/Pt on FTO glass substrate. Fig. 1, Fig. 2, Fig. 3, Fig. 4 show electrochemical catalytic activity performances. Table 1, Table 2, Table 3, Table 4, Table 5 present the adhesion test data, counter electrode properties extracted from the CVs and simulated data of the EIS spectra as calculated from the equivalent circuits.

**Table 1:** CNT-Pt paste behavior.

**Table 2:** Counter electrode properties extracted from the CVs.

CE	Scan rate (mV s^−^^1^)	*V*_oxd_ (V)	*I*_oxd_ (mA)	*V*_red_ (V)	*I*_red_ (mA)
10 mg	50	0.04	0.211	− 0.32	− 0.349
	100	0.07	0.306	− 0.35	− 0.454
	150	0.08	0.384	− 0.36	− 0.525
	200	0.10	0.43	− 0.39	− 0.611
50 mg	50	− 0.03	0.384	− 0.19	− 0.536
	100	0.00	0.588	− 0.21	− 0.721
	150	0.02	0.796	− 0.23	− 0.932
	200	0.04	0.955	− 0.25	− 1.090
100 mg	50	− 0.01	0.49	− 0.2	− 0.605
	100	0.02	0.873	− 0.24	− 1.032
	150	0.05	1.157	− 0.27	− 1.318
	200	0.08	1.398	− 0.29	− 1.564
200 mg	50	− 0.01	0.651	− 0.21	− 0.842
	100	0.03	1.096	− 0.25	− 1.280
	150	0.07	1.487	− 0.28	− 1.658
	200	0.11	1.819	− 0.31	− 1.993

**Table 3:** Simulated data of the EIS spectra as calculated from the equivalent circuits.

CE	*R*_h_ (Ω)	*R*_ct_ (Ω)	W			CPE	
			*R* (Ω)	*T*	*P*	*T*	*P*
10 mg	5.45	8.54	1.67	0.53	0.5	0.00010	0.90
50 mg	4.83	0.75	1.05	0.53	0.5	0.00048	0.98
100 mg	4.86	0.07	1.01	0.56	0.5	0.00100	0.95
200 mg	4.84	0.03	0.53	0.82	0.5	0.00130	0.98

## 2 Experimental design, materials and methods

### 2.1 Materials

Multi-walled carbon nanotube/Pt was purchased from Bioneer Company, Korea. SnO_2_:F (FTO) glass as a conductive transparency electrode was purchased from Pilkington, USA (~ 8 Ω/□). These substrates were used after cleaning by sonic treatment in acetone (Fluka). The electrolyte was a solution of 0.60 M 1-methyl-3-butylimidazolium iodide (Sigma-Aldrich), 0.03 M I_2_ (Sigma-Aldrich), 0.10 M guanidinium thiocyanate (Sigma-Aldrich), and 0.50 M 4-tert-butylpyridine (Aldrich) in a mixed solvent of acetonitrile (Sigma-Aldrich), and valeronitrile, with a volume ratio of 85:15.

**Table 4:** Simulated data of the EIS spectra as calculated from the equivalent circuits.

CE	*R*_h_ (Ω)	*R*_ct_ (Ω)	*W*			CPE	
			*R* (Ω)	*T*	*P*	*T*	*P*
50 mg-300 °C	4.83	0.75	1.05	0.53	0.5	0.00048	0.98
50 mg-400 °C	4.81	0.77	1.07	0.57	0.5	0.00047	0.99

### 2.2 Methods

The fabrication process of making CNT/Pt paste and coating it on FTO glass substrate is shown in Scheme 1. The CNT/Pt pastes with different CNT/Pt content of 10, 50, 100 and 200 mg of CNT/Pt powders in a solution of 40 mg ethyl cellulose (Sigma-Aldrich) in 900 mg alpha-terpineol (Sigma-Aldrich) were prepared through a three-roll miller as described in previous works [1], [2], [3]. To realize the CNT/Pt film-coated FTO glass, the doctor-blade method was applied and the film was dried at 300 °C for 30 min. The developed CEs were denoted as the 10, 50, 100 and 200 mg samples. The properties of the CNT/Pt pastes and CNT/Pt films are presented in Table 1.

### 2.3 Experimental design

The viscosity of the paste is measured by a Brookfield viscometer DV-II pro device. It was found that the viscosity of the CNT/Pt pastes was higher than 1800 cp, making them suitable for the doctor-blade method, as shown in Table 1. The adhesion of the CNT/Pt layers was realized using Scotch tape (Elcometer 107) and was estimated using the ASTM D3359-B standard. The obtained results are presented in Table 1. As shown in the table, the outcomes for all of the formulas were estimated to be close to 5B because the edges of the cuts are completely smooth and none of the squares of the lattice is detached (ASTM D3359-B). No detachment of the CNT/Pt films from FTO glass samples was observed after the adhesion test, suggesting high stability of the CEs in the DSCs.

Fig. 1 shows the CVs of the I^−^/I_3_^−^ system on different CEs at various scan rates (50, 100, 150 and 200 mV s^−^^1^). It was found that the peak current densities changed with a change in the scan rate. Accordingly, the cathodic peaks gradually shifted and became negative while the anodic peaks shifted positively with an increase in the scan rate. As observed in Table 2, linearity in the peak currents (cathodic and anodic) was observed with respect to the square root of the scan rates. These results present evidence of the diffusion limitation of the redox reaction, which may be associated with the desorption of iodide species on the CNT/Pt surface [4], [5], [6]. This phenomenon shows that the adsorption of triiodide species is little affected by the redox reaction on the CE surface, suggesting no specific interaction between the I^−^/I_3_^−^ redox couple and CNT/Pt CEs with different thicknesses.

Table 3 presents the EIS parameters, which were performed on different symmetrical dummy cells fabricated with two identical electrodes. We found that the *R*_*ct*_ values decreased in the order of 10 mg (8.54 Ω) > 50 mg (0.75 Ω) > 100 mg (0.07 Ω) > 200 mg (0.03 Ω). This outcome indicates that the reaction rate of the reduction of the triiodide ions increases with an increase in the amount of CNT/Pt in the paste.

To confirm the effect of annealing temperature, we carried out the EIS measurement for symmetrical dummy cells prepared with different annealing temperatures of 300 and 400 °C. The obtained result is presented in Fig. 2 and Table 4. As can be seen, there is no difference in catalytic activity of two samples prepared by different annealing temperature.

To get insight the effect of thickness, we conducted an experiment with a similar porosity of sample prepared from 50 mg CNT/Pt. The EIS measurement is conducted for this purpose. The obtained results are presented in Fig. 3, Fig. 4 and Table 5. We found that the change in thickness of CNT/Pt strongly affects the electrocatalytic activity of CEs.

**Table 5:** Simulated data of the EIS spectra as calculated from the equivalent circuits.

CE	*R*_h_ (Ω)	*R*_ct_ (Ω)	*W*			CPE	
			*R* (Ω)	*T*	*P*	*T*	*P*
50 mg-3.2 µm thickness	4.83	0.75	1.05	0.53	0.5	0.00048	0.98
50 mg-12 µm thickness	4.75	0.24	1.33	0.97	0.5	0.00068	0.98
